# Sexual Orientation Related Differences in Cortical Thickness in Male Individuals

**DOI:** 10.1371/journal.pone.0114721

**Published:** 2014-12-05

**Authors:** Christoph Abé, Emilia Johansson, Elin Allzén, Ivanka Savic

**Affiliations:** 1 Department of Clinical Neuroscience, Karolinska Institutet, Stockholm, Sweden; 2 Department of Neuroscience, Karolinska Institutet, Stockholm, Sweden; 3 Department of Women's and Children's Health, Division of Pediatric Neurology, Neurology Clinic, Karolinska Hospital, Stockholm, Sweden; University of L'Aquila, Italy

## Abstract

Previous neuroimaging studies demonstrated sex and also sexual orientation related structural and functional differences in the human brain. Genetic information and effects of sex hormones are assumed to contribute to the male/female differentiation of the brain, and similar effects could play a role in processes influencing human's sexual orientation. However, questions about the origin and development of a person's sexual orientation remain unanswered, and research on sexual orientation related neurobiological characteristics is still very limited. To contribute to a better understanding of the neurobiology of sexual orientation, we used magnetic resonance imaging (MRI) in order to compare regional cortical thickness (Cth) and subcortical volumes of homosexual men (hoM), heterosexual men (heM) and heterosexual women (heW). hoM (and heW) had thinner cortices primarily in visual areas and smaller thalamus volumes than heM, in which hoM and heW did not differ. Our results support previous studies, which suggest cerebral differences between hoM and heM in regions, where sex differences have been reported, which are frequently proposed to underlie biological mechanisms. Thus, our results contribute to a better understanding of the neurobiology of sexual orientation.

## Introduction

The origin and development of a person's sexual orientation, and the feeling of being sexually attracted to a person of opposite, same or both sexes, is still a controversial topic. Research pointing to the fact that neurobiological processes are involved in the development of an individual's sexual orientation is emerging. Among the contributing factors it has been suggested that mechanism similar to those influencing the female/male differentiation (sexual dimorphism) of the human brain during the fetal, neonatal and pubertal development could be involved. Such mechanisms include genetic information and/or effects of sex hormones. For example, sexual dimorphism of the brain, as well as sexual orientation has been linked to effects of testosterone, see [1]–[10] and references therein. While the literature on sex differences in the human brain is extensive, research on neurobiological characteristics of sexual orientation in humans is very limited.

### Previous neuroimaging studies

Some previous studies support the hypothesis of a neurobiological basis in the origin of sexual orientation, and have indicated that sexual orientation related structural and functional cerebral differences may exist. See [2], [8] for a comprehensive overview on the topic of sexual orientation related differences in the human brain. In brief, using magnetic resonance imaging (MRI) and positron emission tomography (PET) Savic et al. measured volumetric hemispheric asymmetry and functional connectivity of the amygdala, and found that hemispheric volumes of homosexual men and heterosexual women were symmetric, whereas heterosexual men and homosexual women showed a rightward cerebral asymmetry. In addition, the amygdala connections of the in homosexual men and heterosexual women were found to be more widespread from the left amygdala, whereas heterosexual men and homosexual women the functional connections were more widespread from the right amygdala [11]. Moreover, human pheromone-like compounds activated the anterior hypothalamus of heterosexual women and homosexual men in a similar way, but differently from the subjects of the same sex and opposite sexual orientation [12], [13]. Another MRI study revealed a more male-like structural pattern of grey matter in the perirhinal cortex of homosexual female participants [7]. Furthermore, Kranz et al. found that heterosexual females and homosexual males responded more to photographs of male faces, whereas homosexual females and heterosexual males responded more to photographs showing female faces [14]. It was also reported that compared to heM functional connectivity between a seed region in the left inferior occipital gyrus and the right cuneus was reduced in hoM, and the connectivity between this seed region and the right thalamus and cuneus correlated positively with Kinsey scores [15]. Moreover, during visually evoked sexual arousal different neural circuits were active in hoM and heM: heM showed activation in lingual, hippocampus and parahippocampal gyrus, whereas in hoM the angular gyrus, caudate and pallidum were activated. [16]. Although research pointing to the existence of cerebral differences related to sexual orientation is emerging, knowledge about neuroanatomical characteristics is still poor, and numerous questions about its origin and development still remain unanswered.

### Cortical thickness (Cth)

A metric increasingly used to study brain-behavior relationships in multiple clinical conditions and healthy controls, which is also associated with neurocognitive functions, is cortical thickness (Cth) [17]–[19]. The measure of Cth is related to the size, number and density of cells and dendrites in a cortical column [20], can serve as a proxy marker of the integrity of the cerebral cortex [21]–[23] and was shown to be related to brain metabolite concentrations, such as N-acetylaspartate [23]. The genetic coding of Cth seems also to be independent from the genetic coding of cortical surface area and volume [24], [25]. Intriguingly, Bramen et al. found that Cth was associated to circulating testosterone levels differently in adolescent boys (positively) and girls (negatively), and predominantly in regions high in androgen receptors, such as the medial occipital cortex [26]. Furthermore, the corresponding correlation seems to become inversed in the adulthood [27], [28] illustrating the complexity of the sex hormone interaction with sex, stage of development and region of the brain. Nevertheless, the reported preliminary observations are of interest for the research of sexual orientation, since testosterone is believed to have impact on sex differences in brain structure and some functions [29]–[31] implicating a possible link also to sexual orientation [2], [9], [32], [33]. In this respect it is also of note that sex differences in Cth have been reported, primarily in frontal, parietal and occipital areas of the brain [28], [34]–[39], albeit with some inconsistencies between the still limited reports in the literature. For example, using brain size normalized data Luders et al. demonstrated higher cortical thickness in females compared to males wide spread over all lobes, whereas when analyzing unscaled data females had thicker cortices in e.g. lateral superior pre- and postcentral regions, the occipital lobe and in left frontal gyrus. In addition, they showed that males had thicker cortices than females in a region of the left lateral temporal lobe, as also found by Lv et al [38]. Furthermore, Sowell et al. found thicker cortices in a region comprising the right inferior parietal and posterior temporal regions of females compared to males, when not correcting for differences in brain size, and males had thicker cortices than females in right anterior temporal and orbitofrontal regions [34]. In another study, Im et al. demonstrated greater cortical thickness in females in superior parietal and postcentral gyrus, but in contrast to other studies locally thicker cortices in men were not observed [37]. Presumably, some of these inconsistencies may stem from population based and/or methodological differences across studies, and taken together, further *in vivo* study is required to elucidate the relationship between sex and regional Cth.

### The present study

Findings in previous studies suggest that certain cerebral characteristics might differ between homosexual and heterosexual individuals, and that homosexual individuals may have some cerebral features, that are more similar to their opposite sex heterosexual counterpart. Moreover, sex differences in cortical thickness were reported, and testosterone effects are assumed to play a role in sexual dimorphism of the brain, as well as being linked to the development of a certain sexual orientation. Thus, based on previous studies we hypothesized that sexual orientation related differences in regional Cth could be found, most likely in regions considered as sexually dimorphic (reviewed above) and/or regions high in androgen receptors, such as the medial occipital lobe.

To our knowledge, no previous studies investigated Cth differences in relation to sexual orientation. In pursuit of a better understanding of the origin and development of sexual orientation and to expand the knowledge about sexual orientation specific differences in the human brain, we investigated differences in regional cortical thickness between heterosexual men (heM) and homosexual men (hoM). To further explore whether potential differences are present in regions, where sex specific differences have been reported previously, we also compared both groups to heterosexual women (heW).

## Methods

### Participants

Sixty one healthy age-matched hoM (n = 19), heM (n = 21) and heW (n = 21) were recruited from the local community. The Ethics Committee of the Karolinska Institutet Stockholm approved the study, and prior to study all participants signed informed consent forms. heM and hoM differed from heW in biological sex (male/female), whereas heM and hoM only differed in sexual orientation. Sexual orientation was assessed through the Kinsey hetero/homosexual self-identification scale (0: exclusively heterosexual, 6: exclusively homosexual) [40]. Individuals were considered as heterosexual when scoring between 0 and 1, and as homosexual when scoring between 5 and 6 on the Kinsey scale. Participants reported no history of psychiatric disorders, head trauma and head injury resulting in loss of consciousness for more than 10 minutes, HIV/AIDS, Hepatitis-C, chronic pain conditions, vision or hearing problems. In addition, all MRI scans were evaluated by a clinical radiologist to assure that the analyzed cohort show no brain pathology. To assure a balanced number of left and right handed participants in each group handedness was determined using the Edinburgh Handedness Inventory [41]. Participants' characteristics are described in more detail in the results section.

### MRI acquisition

3D T1-weighted SPGR (spoiled gradient echo; TE  = 3.1 ms, TR  = 7.9 ms, TI  = 450 ms, FOV  = 24 cm, matrix = 240×240, 176 axial slices, flip angle  = 12 degree) were acquired with 1 mm^3^ isotropic resolution using a 3-Tesla MRI medical scanner (Discovery 3T GE-MR750, General Electric, Milwaukee, Wisconsin) equipped with an 8-channel phased array receiving coil.

### Image processing

Cortical thickness (Cth) measures were obtained using the semi-automated subcortical volumetric segmentation and cortical surface reconstruction methods provided by Freesurfer v5.1. Methodological details of the individual processing steps can be found in [42]–[47]. In brief, the procedure includes intensity normalization, removal of non-brain tissue, segmentation of cortical gray, subcortical white, deep gray matter volumetric structures, triangular tessellation of the gray/white matter interface and the pial surface (white matter/cerebrospinal fluid boundary). Cortical thickness is calculated as the closest distance from the gray/white to the pial surface at each vertex on the reconstructed surfaces. All reconstructions were visually inspected and, where necessary, manual corrections were made by trained operators, including corrections of erroneous skull striping, white matter, grey matter and subcortical segmentation using editing tools provided by Freesurfer. Freesurfer allows an automated parcellation of the cortical surfaces into 34 anatomical regions of interest (ROI) for each hemisphere, segmentation of subcortical ROIs (thalamus, caudate, putamen, hippocampus, amygdala) and cerebellum, see [45], [48] for technical details. For each ROI, Cth and subcortical volume measures were obtained from subjects' native space.

### Statistical analyses

Overall group differences in regional Cth and subcortical volumes were tested in separate univariate analyses of covariance (ANCOVA) using SPSS v20, with the MRI outcome measure as dependent variable, age as covariate and group (heM, hoM and heW) as fixed factor. Significant ANCOVA findings for group (p<0.05) were followed up by pairwise group comparisons (t-tests) to understand the nature of the main effect for the group factor. Since the tested outcome measures are correlated to each other, we corrected univariate and pairwise group analyses for multiple comparisons with an adjusted (or partial) Bonferroni method, details on the applied Dubey/Armittage-Parmaris procedure can be found in [49]. Alpha levels (0.05) were adjusted to p = 0.0024 (cortical) and p = 0.0133 (subcortical) using the average inter correlation coefficients among all investigated cortical (r = 0.28) and subcortical ROIs (r = 0.46) for the combined cohort. Those adjusted alpha levels represent the upper boundaries of p values, which can be considered as statistically significant after correction for multiple comparisons. Effect sizes for group mean differences were calculated via Cohen's d. We also tested for effects of years of education and total brain volume, respectively, by entering those variables simultaneous with age as covariates. Before final analysis, distributions of all investigated variables were tested for normality using the one-sample Kolmogorov-Smirnov test within each group separately and in the combined group. We also analyzed sexual orientation related differences in Cth on a vertex level. The methods and results of this secondary analysis are presented in File S1 .

## Results

### Demographic variables

Groups did not differ in age (heM: 31.9±6.0 years, heW: 33.2±6.1 years, hoM: 33.5±6.1 years), years of education (heM: 15.9±2.0, heM: 17.3±3.3, hoM: 16.4±2.9) or handedness scores (heM: 74±26, heW: 77±26, hoM: 85±27) and participants were primarily right handed. Two participants in the female group had slightly negative scores of −6 and −20, respectively (mixed-handedness). Heterosexual individuals scored in average 0.0±0.5, and homosexual individuals 5.5±0.5 on the Kinsey scale.

### Group comparisons of Cth and subcortical volumes

Univariate tests were significant for group differences in Cth in the left middle and superior temporal ROI, in the right inferior temporal, lateral orbitofrontal, pars triangularis, lingual, cuneus and pericalcarine ROI (all 0.0001<p<0.041). Group differences were also significant for subcortical volumes in amygdala, putamen, thalamus and cerebellum (all 0.0001<p<0.004). Results of pairwise group statistics of regions in which group differences were found are shown in Table 1 and 2. MR measures (group means of Cth and subcortical volumes) of all investigated ROIs can be found in Table S1 and Table S2. In pairwise group comparisons of Cth in the left hemisphere, heW demonstrated significantly thinner cortices than hoM in the middle and superior temporal ROI. The same was observed comparing heW to heM, where the difference in the middle temporal ROI (p = 0.004) can be considered as trend after Bonferroni adjustment. No differences were found between heM and hoM. In the right hemisphere, cortices of heW and hoM were thinner compared to heM in the lateral orbitofrontal, as well as in the lingual, pericalcarine and cuneus ROI, where the differences in right lateral orbitofrontal (p = 0.003) and cuneus (p = 0.003) are considered as strong trends after Bonferroni adjustments. In those ROIs no differences were found between heW and hoM. In addition, hoM showed thinner cortices than heM (and same trend compared to heW) in the right pars triangularis and inferior temporal ROI. Where significant differences or trends (p<0.1) were found effect sizes were moderate (0.5–0.79) to strong (≥0.80) [50].

**Table 1:** Group differences in Cth.

hemisphere	ROI	heM	heW	hoM	ANCOVA, p	heM vs. heW, p (ES)	heM vs. hoM, p (ES)	heW vs. hoM, p (ES)	pattern
left	middle temporal	2.92±0.13	2.81±0.12	2.95±0.13	0.002*	**0.004 (0.91)**	NS (0.20)	0.001* (1.13)	heW <both
	superior temporal	2.89±0.12	2.79±0.12	2.87±0.12	0.022	**0.009 (0.83)**	NS (0.17)	**0.038 (0.67)**	heW <both
right	inferior temporal	2.8±0.16	2.75±0.16	2.67±0.16	0.041	NS (0.33)	**0.009 (0.86)**	0.096 (0.54)	hoM <both
	lateral orbitofrontal	2.77±0.12	2.65±0.12	2.66±0.12	0.004	**0.003 (0.97)**	**0.006 (0.92)**	NS (0.06)	heM> both
	pars triangularis	2.51±0.12	2.49±0.12	2.38±0.12	0.005	NS (0.16)	0.002* (1.01)	**0.009 (0.85)**	hoM <both
	lingual	2.30±0.12	2.07±0.12	2.09±0.12	<0.001*	<0.001* (1.91)	<0.001* (1.76)	NS (0.17)	heM> both
	cuneus	2.12±0.15	1.99±0.14	1.96±0.14	0.001*	**0.003 (0.95)**	0.001* (1.14)	NS (0.20)	heM> both
	pericalcarine	1.86±0.19	1.65±0.19	1.57±0.19	<0.001*	0.001* (1.08)	<0.001* (1.52)	NS (0.44)	heM> both

Regional Cth (mean ± standard deviation, in mm, the values are adjusted for age), results (p-values) of pairwise group comparisons and corresponding effect sizes (ES). Significant p-values (p<0.05) are listed in bold and trends (p<0.1) in normal font. P-values surviving multiple comparison corrections (Bonferroni adjusted p = 0.0024) are highlighted by *. NS: not significant.

**Table 2:** Group differences in subcortical volumes.

hemisphere	ROI	heM	heW	hoM	ANCOVA p	heM vs. heW, p (ES)	heM vs. hoM, p (ES)	heW vs. hoM, p (ES)	pattern
left	amygdala	1973±270	1679±265	2019±265	<0.001*	0.001* (1.10)	NS **(0.17)**	<0.001* (1.23)	heW <both
	putamen	5262±549	4623±549	5228±549	<0.001*	<0.001* (1.16)	NS **(0.06)**	0.001* (1.10)	heW <both
	thalamus	7887±604	7148±604	6915±601	<0.001*	<0.001* (1.22)	<0.001* (1.61)	**NS (0.39)**	heM> both
	hippocampus	4312±513	3923±513	4021±514	0.048	**0.018** **(0.76)**	0.081 (0.57)	NS (0.19)	heM> both
	cerebellum	74041±5237	65144±5219	73999±5226	<0.001*	<0.001* (1.70)	NS **(0.01)**	<0.001* (1.70)	heW <both
right	amygdala	2102±261	1875±261	2130±261	0.004*	0.007* (0.87)	NS **(0.11)**	0.003* (0.98)	heW <both
	putamen	5166±531	4464±531	4895±531	<0.001*	<0.001* (1.32)	NS **(0.51)**	0.013* (0.81)	heW <both
	thalamus	7930±604	7019±604	6780±601	<0.001*	<0.001* (1.51)	<0.001* (1.91)	NS **(0.40)**	heM> both
	hippocampus	4337±362	4114±362	4357±361	0.064	**0.050** **(0.62)**	NS (0.06)	**0.037** **(0.67)**	heW <both
	cerebellum	76315±5906	67068±5884	75786±5888	<0.001*	<0.001* (1.57)	NS **(0.09)**	<0.001* (1.48)	heW <both

Regional subcortical volumes (mean ± standard deviation, in mm^3^) adjusted for age. Results (p-values) of pairwise group comparisons and corresponding effect sizes (ES) are shown. Significant p-values (p<0.05) are listed in bold and trends (p<0.1) in normal font. P-values surviving multiple comparison corrections (Bonferroni adjusted p = 0.0133) are highlighted with *. NS: not significant. Results of secondary analysis correcting for brain volumes are described in the text.

heW had smaller volumes than both, heM and hoM, in left and right amygdale, putamen and cerebellum. However, in both hemispheres thalamus volumes of both hoM and heW were smaller than in heM, whereas thalamus volumes of hoM and heW did not differ. Controlling for years of education and brain volume, respectively, did not change the reported results of group differences in Cth. The same applies for group comparisons of subcortical volumes, with the exemption: when correcting for brain volume, groups were similar in amygdale, thalamus and putamen volumes, but hoM had still significantly smaller thalamus volumes than heM and heW (all p<0.001). Excluding the two ambidextrous female participants from the analysis did not change the results for observed group differences, neither in Cth nor subcortical volumes.

## Discussion

To contribute to a better understanding of the neurobiology of sexual orientation, we investigated possible differences in regional cortical thickness (Cth) between heterosexual men (heM) and homosexual men (hoM). The group comparisons included heterosexual women (heW) in order to explore, whether (and if so, which of) the differences observed were present in regions, which indicate sex related differences.

**Figure 1:**
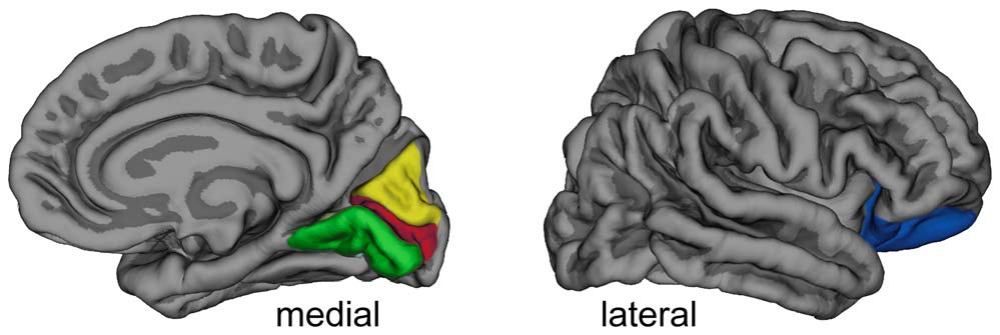
Sex and sexual orientation related differences in Cth. Freesurfer parcellated cortical ROIs of the right hemisphere in which a pattern of sex and sexual orientation related differences in Cth were found are highlighted (heM> both, hoM and heW; with heW  =  hoM). Yellow: cuneus, red: pericalcarine, green: lingual and blue: lateral orbitofrontal ROI.

Comparing heM and hoM, it was found that hoM had thinner cortices compared to heM in the right lateral orbitofrontal ROI, as well as in ROIs located in the right visual cortex (lingual, pericalcarine and cuneus). The same regions also showed thinner cortex in heW than in heM, whereas no differences were found between heW and hoM (see Figure 1). The additional sex related differences revealed in the left temporal and right orbitofrontal regions are in agreement with what was found in previous studies [34], [36], [38], [39]. Some previous studies of sex differences in Cth reported thicker cortices in women in a right temporoparietal region [28], [34] or a lateral superior pre- and postcentral region [28], [36], which was not found in this study. One possible reason for that could be that the ROI analysis used here is restricted to Cth measures averaged within a single predefined region. Consequently, potential differences localized in a relatively small ROI subregion or in a region comprised by small ROI subregions will not be detected by the presently used approach. In addition, when comparing male and female brains, which differ in size, group differences might be less pronounced when not correcting for brain volumes [36]. However, by analyzing Cth measures obtained from the individual brain's native space the observed group differences have the advantage of not being influenced by scaling and normalization procedures, although this is at the cost of the spatial resolution of areas showing possible group differences. Another potential explanation for the described discrepancies compared with some previous studies is that sexual orientation of study participants, which could have influenced the results, was not considered in some of these previous studies. heM and hoM did not differ in brain volumes, and yet the sexual orientation related differences in Cth were similar to the here indicated sex related differences, which revealed strong effect sizes. These results could suggest that hoM differ from heM in regions, in which previous studies found sex related differences, and/or revealed high density of androgen receptors. More specifically, the present results indicate that hoM have different cortical thickness compared with heM in areas of the medial occipital lobe.

The underlying mechanisms leading to the observed structural differences related to sexual orientation are unknown, and cannot be derived from this study. One possibility is that biological processes frequently proposed to underlay sex differences, such as gene and sex hormone dependent mechanisms (e.g. testosterone effects) during pre- and postnatal development, may interact with the cortical architecture in visual areas resulting in different Cth in hoM compared to heM. For example, a recent study has shown that in visual areas (especially the right lingual gyrus), reported to be dense in androgen receptors, higher circulating testosterone levels were associated with thinner cortices in girls, but with thicker cortices in boys during adolescence [26]. The authors suggested that the positive correlations observed in boys could represent an effect of testosterone on cortical thickening by stimulating neurogenesis, or reflect a preservation of brain tissue by blocking apoptosis in visual areas, whereas in girls opposite effects may be operating. However, if or to what degree testosterone effects contribute to the cortical architecture in hoM, heM and heW is an open question, especially since associations between testosterone and Cth are complex and vary depending of brain region, sex and age [27]. Also, considering the fact that hoM had thinner cortices than both heM and heW in the right pars triangularis and the inferior temporal ROI, suggests that cerebral differences related to male homosexuality might be present in regions, which are not necessarily considered as sexually dimorphic. Thus, more research is needed to investigate the structural underpinnings of sexual orientation.

It is also of interest that the thalamus volume was found to be smaller in hoM than in both heM and heW (see Table 2). Functional connectivity involving the right thalamus and the right cuneus were reported to be different between hoM and heM, and even showed correlations with Kinsey scale scores [15]. Furthermore, the thalamus is reported to be involved in sexual arousal and reward processes; During visually evoked sexual arousal both heM and hoM activated the thalamus, but heM, showed, in contrast to hoM, also activation in the lingual gyrus [16]. Hence, the here reported sexual orientation related structural differences in the visual cortex and thalamus support other studies suggesting sexual orientation related characteristics in visual perception and processing, and point to possible differences in other cognitive processes, in which visual areas are recruited (e.g. working memory, mental imagery and auditory attention [51]–[57].

In summary, the present study is the first to indicate sexual orientation related differences in regional Cth. It indicates similar cortical structure in heW and hoM in early visual areas, which are different from heM. The results add to the existing literature on sex and sexual orientation related structural differences of the cerebral cortex, and, therefore, contribute to a better understanding of the neurobiology of sexual orientation. In addition, the data suggest that sexual orientation of study participants should be gathered in neuroimaging studies, since their characteristics may bias neuroimaging findings.

### Study limitations and Outlook

The present findings relate only to male homosexuality, and the generated data cannot be extrapolated to female homosexuality, which requires separate studies to be conducted. The data consisted of average thickness values obtained from relatively large cortical regions defined by the Desikan's atlas. Thus evaluations of group comparisons with higher regional resolution (e.g. vertex-wise analysis), as well as investigations of larger populations will most likely give more detailed and/or additional information about sexual orientation related cortical characteristics.

Furthermore, participants did not report any history of psychiatric disorders; however, we did not administer depression, mood or anxiety measures, which may be related to the morphological measures. Furthermore, differences in body mass index, diet and exercise, as well as smoking status may have influenced the findings. Also here, larger study samples are needed to identify the degree to which those factors affect our results. Although the present data do not provide information about the underlying mechanisms, and may not disentangle genetic, hormonal or other environmental processes, they open a direction for future investigations of the biology of sexual orientation.

## Supporting Information

Table S1 **Descriptive group data of cortical ROIs.** Cortical thickness obtained for heM, heW and hoM (mean ± standard deviation) in all Freesurfer parcellated ROIs.
(DOCX)

Table S2 **Descriptive group data of subcortical ROIs.** Subcortical volumes in all ROIs obtained for heM, heW and hoM (mean ± standard deviation).
(DOCX)

File S1 **Vertex-wise analysis of sexual orientation related differences in Cth.**
(DOCX)
