# Supplementary material for: Sexual Orientation Related Differences in Cortical Thickness in Male Individuals
Source: PLoS One. 2014 Dec 5;9(12):e114721. doi: 10.1371/journal.pone.0114721 (PMC4257718; doi:10.1371/journal.pone.0114721)
Supplement: Table S1 — Descriptive group data of cortical ROIs. Cortical thickness obtained for heM, heW and hoM (mean ± standard deviation) in all Freesurfer parcellated ROIs. (DOCX) [file pone.0114721.s001.docx]

|  | heM | | heW | | hoM | |
| --- | --- | --- | --- | --- | --- | --- |
| ROI | left hemisphere | right hemishpere | left hemisphere | right hemishpere | left hemisphere | right hemishpere |
| banks sts | 2.45 ± 0.18 | 2.55 ± 0.21 | 2.33 ± 0.14 | 2.59 ± 0.16 | 2.41 ± 0.20 | 2.56 ± 0.17 |
| caudal anterior cingulate | 3.05 ± 0.14 | 2.80 ± 0.26 | 3.00 ± 0.24 | 2.81 ± 0.29 | 2.99 ± 0.20 | 2.86 ± 0.25 |
| caudal middle frontal | 2.70 ± 0.16 | 2.61 ± 0.11 | 2.67 ± 0.18 | 2.60 ± 0.10 | 2.63 ± 0.17 | 2.54 ± 0.13 |
| cuneus | 2.01 ± 0.16 | 2.13 ± 0.19 | 2.01 ± 0.15 | 1.98 ± 0.13 | 2.05 ± 0.12 | 1.95 ± 0.12 |
| entorhinal | 3.31 ± 0.28 | 3.56 ± 0.39 | 3.37 ± 0.35 | 3.54 ± 0.38 | 3.36 ± 0.34 | 3.43 ± 0.35 |
| fusiform | 2.76 ± 0.12 | 2.83 ± 0.16 | 2.73 ± 0.11 | 2.82 ± 0.11 | 2.74 ± 0.13 | 2.76 ± 0.12 |
| inferior parietal | 2.45 ± 0.11 | 2.65 ± 0.12 | 2.44 ± 0.11 | 2.62 ± 0.12 | 2.47 ± 0.12 | 2.61 ± 0.13 |
| inferior temporal | 2.75 ± 0.14 | 2.80 ± 0.14 | 2.68 ± 0.15 | 2.75 ± 0.16 | 2.74 ± 0.13 | 2.67 ± 0.18 |
| isthmuscingulate | 2.72 ± 0.16 | 2.76 ± 0.15 | 2.72 ± 0.23 | 2.65 ± 0.18 | 2.76 ± 0.19 | 2.73 ± 0.20 |
| lateral occipital | 2.16 ± 0.15 | 2.42 ± 0.17 | 2.11 ± 0.09 | 2.34 ± 0.12 | 2.14 ± 0.11 | 2.34 ± 0.14 |
| lateral orbitofrontal | 2.79 ± 0.16 | 2.78 ± 0.13 | 2.75 ± 0.17 | 2.65 ± 0.14 | 2.79 ± 0.12 | 2.65 ± 0.15 |
| lingual | 2.15 ± 0.10 | 2.30 ± 0.15 | 2.10 ± 0.10 | 2.07 ± 0.11 | 2.09 ± 0.12 | 2.09 ± 0.09 |
| medial orbitofrontal | 2.66 ± 0.16 | 2.74 ± 0.32 | 2.64 ± 0.16 | 2.58 ± 0.19 | 2.65 ± 0.11 | 2.64 ± 0.18 |
| middle temporal | 2.93 ± 0.13 | 2.81 ± 0.18 | 2.80 ± 0.12 | 2.86 ± 0.17 | 2.94 ± 0.15 | 2.77 ± 0.17 |
| parahippocampal | 2.88 ± 0.31 | 2.75 ± 0.25 | 2.87 ± 0.28 | 2.81 ± 0.20 | 2.78 ± 0.37 | 2.82 ± 0.31 |
| paracentral | 2.58 ± 0.16 | 2.54 ± 0.14 | 2.54 ± 0.12 | 2.53 ± 0.12 | 2.51 ± 0.14 | 2.54 ± 0.14 |
| pars opercularis | 2.67 ± 0.15 | 2.57 ± 0.10 | 2.65 ± 0.11 | 2.58 ± 0.15 | 2.66 ± 0.12 | 2.51 ± 0.13 |
| pars orbitalis | 2.80 ± 0.29 | 2.68 ± 0.17 | 2.78 ± 0.21 | 2.67 ± 0.14 | 2.82 ± 0.19 | 2.67 ± 0.17 |
| pars triangularis | 2.64 ± 0.20 | 2.51 ± 0.15 | 2.57 ± 0.11 | 2.48 ± 0.12 | 2.58 ± 0.16 | 2.38 ± 0.12 |
| pericalcarine | 1.79 ± 0.18 | 1.86 ± 0.23 | 1.78 ± 0.14 | 1.65 ± 0.16 | 1.82 ± 0.13 | 1.57 ± 0.16 |
| postcentral | 2.18 ± 0.12 | 2.17 ± 0.08 | 2.14 ± 0.11 | 2.13 ± 0.11 | 2.13 ± 0.11 | 2.12 ± 0.11 |
| posterior cingulate | 2.86 ± 0.09 | 2.77 ± 0.17 | 2.80 ± 0.16 | 2.80 ± 0.13 | 2.76 ± 0.16 | 2.78 ± 0.15 |
| precentral | 2.66 ± 0.11 | 2.59 ± 0.09 | 2.60 ± 0.10 | 2.56 ± 0.09 | 2.62 ± 0.11 | 2.54 ± 0.11 |
| precuneus | 2.50 ± 0.10 | 2.59 ± 0.13 | 2.46 ± 0.09 | 2.55 ± 0.09 | 2.49 ± 0.10 | 2.61 ± 0.11 |
| rostral anterior cingulate | 3.14 ± 0.22 | 2.98 ± 0.34 | 3.05 ± 0.26 | 2.94 ± 0.27 | 3.02 ± 0.15 | 3.06 ± 0.15 |
| rostral middle frontal | 2.63 ± 0.16 | 2.36 ± 0.10 | 2.53 ± 0.15 | 2.32 ± 0.08 | 2.57 ± 0.12 | 2.33 ± 0.10 |
| superior frontal | 2.95 ± 0.13 | 2.80 ± 0.12 | 2.88 ± 0.12 | 2.77 ± 0.12 | 2.88 ± 0.11 | 2.79 ± 0.11 |
| superior parietal | 2.30 ± 0.11 | 2.38 ± 0.11 | 2.28 ± 0.09 | 2.38 ± 0.10 | 2.29 ± 0.08 | 2.35 ± 0.09 |
| superior temporal | 2.89 ± 0.11 | 2.70 ± 0.17 | 2.78 ± 0.11 | 2.73 ± 0.16 | 2.86 ± 0.15 | 2.70 ± 0.14 |
| supramarginal | 2.58 ± 0.11 | 2.62 ± 0.14 | 2.53 ± 0.13 | 2.61 ± 0.11 | 2.54 ± 0.15 | 2.61 ± 0.08 |
| frontal pole | 2.90 ± 0.20 | 2.75 ± 0.27 | 2.91 ± 0.33 | 2.83 ± 0.27 | 2.90 ± 0.32 | 2.84 ± 0.28 |
| temporal pole | 3.67 ± 0.36 | 3.50 ± 0.51 | 3.60 ± 0.31 | 3.70 ± 0.34 | 3.74 ± 0.28 | 3.75 ± 0.31 |
| transverse temporal | 2.58 ± 0.20 | 2.54 ± 0.19 | 2.53 ± 0.15 | 2.48 ± 0.12 | 2.47 ± 0.20 | 2.44 ± 0.14 |
| insula | 3.09 ± 0.15 | 3.09 ± 0.20 | 3.09 ± 0.14 | 3.07 ± 0.09 | 3.08 ± 0.17 | 3.01 ± 0.16 |

Table S1: **Descriptive group data of cortical ROIs.** Cortical thickness obtained for heM, heW and hoM (mean ± standard deviation) in all Freesurfer parcellated ROIs.
