# Supplementary material for: Sexual Orientation Related Differences in Cortical Thickness in Male Individuals
Source: PLoS One. 2014 Dec 5;9(12):e114721. doi: 10.1371/journal.pone.0114721 (PMC4257718; doi:10.1371/journal.pone.0114721)
Supplement: Table S2 — Descriptive group data of subcortical ROIs. Subcortical volumes in all ROIs obtained for heM, heW and hoM (mean ± standard deviation). (DOCX) [file pone.0114721.s002.docx]

| \|  \| heM \| \| heW \| \| hoM \| \| \| --- \| --- \| --- \| --- \| --- \| --- \| --- \| \| ROI \| left hemisphere \| right hemishpere \| left hemisphere \| right hemishpere \| left hemisphere \| right hemishpere \| \| amygdala \| 1979 ± 247 \| 2103 ± 276 \| 1677 ± 242 \| 1875 ± 228 \| 2016 ± 312 \| 2130 ± 266 \| \| hippocampus \| 4318 ± 394 \| 4351 ± 357 \| 3920 ± 499 \| 4108 ± 365 \| 4017 ± 627 \| 4347 ± 384 \| \| caudate \| 4478 ± 595 \| 4504 ± 623 \| 4155 ± 460 \| 4171 ± 494 \| 4360 ± 598 \| 4355 ± 603 \| \| putamen \| 5290 ± 572 \| 5188 ± 478 \| 4612 ± 562 \| 4455 ± 514 \| 5209 ± 580 \| 4880 ± 638 \| \| thalamus \| 7908 ± 601 \| 7957 ± 562 \| 7140 ± 648 \| 7009 ± 583 \| 6901 ± 579 \| 6762 ± 713 \| \| cerebellum \| 74273 ± 5206 \| 76584 ± 6999 \| 65054 ± 5405 \| 66963 ± 5525 \| 73844 ± 5521 \| 75605 ± 5532 \| |  |  |  |  |  |  |  |
| --- | --- | --- | --- | --- | --- | --- | --- | --- | --- | --- | --- | --- | --- | --- | --- | --- | --- | --- | --- | --- | --- | --- | --- | --- | --- | --- | --- | --- | --- | --- | --- | --- | --- | --- | --- | --- | --- | --- | --- | --- | --- | --- | --- | --- | --- | --- | --- | --- | --- | --- | --- | --- | --- | --- | --- | --- | --- | --- | --- | --- | --- | --- | --- |

Table S2: **Descriptive group data of subcortical ROIs.** Subcortical volumes in all ROIs obtained for heM, heW and hoM (mean ± standard deviation).
