# Supplementary material for: Sexual Orientation Related Differences in Cortical Thickness in Male Individuals
Source: PLoS One. 2014 Dec 5;9(12):e114721. doi: 10.1371/journal.pone.0114721 (PMC4257718; doi:10.1371/journal.pone.0114721)
Supplement: File S1 — Vertex-wise analysis of sexual orientation related differences in Cth. (DOCX) [file pone.0114721.s003.docx]

Sexual Orientation Related Differences in Cortical Thickness in Male Individuals

**Supporting information S1: Sexual orientation related differences in cortical thickness analyzed on a vertex level**

**Method:** Sexual orientation related differences in cortical thickness (Cth) were also performed on a vertex level using the QDEC tool provided by Freesurfer. Individual reconstructed surfaces were smoothed, transformed and resampled onto a common standard space (fsaverage) using the

-qcache command (surface-based smoothing FWHM=10mm). Age was used as covariate. Correction for multiple comparison was done using a Monte Carlo cluster-wise simulation (treshold = 1.3, p=0.05).


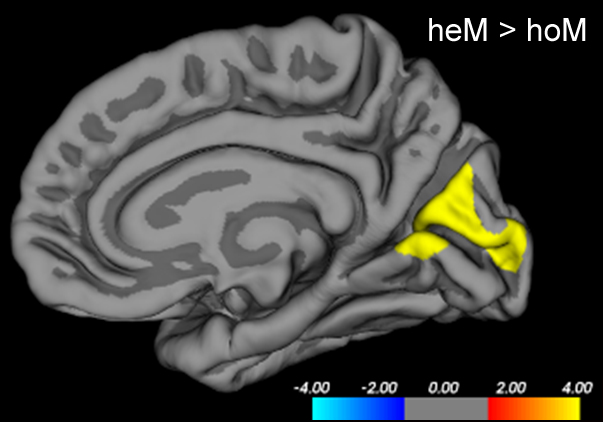


**Figure S1:** The obtained significance of the heM > hoM cluster (yellow) obtained after correction for multiple comparisons is 4.00 (equivalent to p=0.0001). No differences were found in the left hemisphere.

**Results:** Significant differences were found in the right medial occipital lobe, in which heM showed greater cortical thickness than hoM (see Figure S1). The cluster included parts of the lingual, cuneus and pericalcarine, which is in very good agreement with the results obtained from the ROI analysis.

The ROI analysis revealed thicker cortices of heM compared to hoM in the right orbitofrontal cortex, which was not found in the QDEC analysis. On one hand, this might indicate a lack of statistical power for the Monte Carlo cluster-wise simulation approach used for multiple comparison correction in the QDEC analysis. On the other hand, however, weaker and more diffuse effects could be present in the orbitofrontal region, which might be better captured by the ROI approach than in a vertex-wise analysis.

In summary, the results from the vertex wise analysis were largely in agreement with the ROI analysis, and they further indicate sexual orientation related differences in the right medial occipital lobe.
